# Effects of Lornoxicam on Anastomotic Healing: A Randomized, Blinded, Placebo-Control Experimental Study

**DOI:** 10.1155/2016/4328089

**Published:** 2016-04-07

**Authors:** Stamatoula Drakopoulou, Elissaios Kontis, Eirini Pantiora, Antonios Vezakis, Despoina Karandrea, Eftychia Aravidou, Agathi Konti-Paphiti, Erifili Argyra, Dionisios Voros, Andreas A. Polydorou, Georgios P. Fragulidis

**Affiliations:** ^1^2nd Department of Surgery, “Aretaieio” Hospital, National and Kapodistrian University of Athens, School of Medicine, 11528 Athens, Greece; ^2^Department of Pathology, “Aretaieio” Hospital, National and Kapodistrian University of Athens, School of Medicine, 11528 Athens, Greece; ^3^Animal House Facility/Experimental Unit, “Aretaieio” Hospital, National and Kapodistrian University of Athens, School of Medicine, 11528 Athens, Greece; ^4^1st Department of Anesthesiology, “Aretaieio” Hospital, National and Kapodistrian University of Athens, School of Medicine, 11528 Athens, Greece

## Abstract

*Introduction and Aim*. With the implementation of multimodal analgesia regimens, Nonsteroidal Anti-Inflammatory Drugs (NSAIDs) are often administered for optimal pain control and reduction of opioid use. The aim of the study was to examine the effects of lornoxicam, a NSAID, on anastomotic healing employing an animal model.* Materials and Methods*. A total of 28 Wistar rats were randomly assigned in two groups. All animals underwent ascending colonic transection followed by an end-to-end hand sewn anastomosis. Group 1 received intraperitoneally lornoxicam before and daily after surgery. Group 2 received intraperitoneally an equal volume of placebo. Half of the animals in each group were euthanized on the 3rd pod and the remaining on the 7th pod. Macro- and microscopic indicators of anastomotic healing were compared using a two-tailed Fisher exact test.* Results*. The lornoxicam group significantly decreased fibroblast in growth and reepithelization of the mucosa at the anastomotic site on the 3rd pod and significantly increased occurrence of deep reaching defects, necrosis, and microabscess on the 7th pod.* Conclusion*. Lornoxicam administration during the perioperative period adversely affects histologic parameters of intestinal anastomotic healing. These effects of lornoxicam administration were not found to induce significant increase of anastomotic dehiscence in the rat model.

## 1. Introduction

Anastomotic dehiscence following colorectal surgery is a dreaded clinical complication affecting up to 10% of patients and is associated with considerable morbidity, mortality, and even increased risk of local recurrence of cancer [1–4]. Various factors have been implicated in anastomotic healing and reduction of risk factors is a priority for gastrointestinal surgeons [3, 4]. Following identification of increased cardiovascular and gastrointestinal complications with Nonsteroidal Anti-Inflammatory Drugs (NSAIDs) in nonsurgical patients, there has been a growing interest for a possible association between NSAIDs and intestinal anastomotic failure [5–8]. NSAIDs have gained interest in gastrointestinal surgery by the implementation of colonic fast-track surgery, where optimal pain control is warranted to allow early mobilization [9, 10]. Epidural analgesia and NSAIDs are recommended within the Enhanced Recovery After Surgery (ERAS) care protocol as modes to obtain optimal pain control and reduce opioid usage, thus decreasing their side-effects and enhancing early bowel function recovery [11]. NSAIDs inhibit Cyclooxygenase (COX) isoenzymes that play a pivotal role in the arachidonic acid pathway resulting in inhibition of prostaglandin synthesis [12]. However, COX inhibitors may also adversely affect anastomotic healing due to their inhibitory effect on the inflammation process, which is an integral part of wound healing. The perioperative ERAS care after elective rectal and colonic surgery has identified a possible link between NSAIDs and anastomotic leak [9].

Thereafter, numerous clinical and experimental studies regarding the effects of NSAIDs administration on newly constructed intestinal anastomosis have reported controversial results. However there is lack of compelling evidence to abolish usage of NSAIDs during the immediate postoperative period, until further studies addressing this question have been carried out [13]. To this end, we carried out a study to investigate the effects of perioperative lornoxicam usage, a nonselective COX inhibitor, in anastomotic healing in an experimental model.

## 2. Materials and Methods

This study protocol was approved by the ethical committees of the 2nd Department of Surgery “Aretaieio” Hospital University of Athens School of Medicine and the Veterinary Services of Prefecture of Athens (protocol number: 1581/08-03-2012). Twenty-eight male Wistar rats, weighing between 280 and 350 g, were included in the study. Animals were housed two per cage and accustomed to laboratory conditions, in accordance with European Commission Directive 86/609/EEC for animal experiments, at the Experimental Unit of the 2nd Department of Surgery “Aretaieio” Hospital University of Athens. Standard laboratory diet and water were administered ad libitum pre- and postoperatively.

### 2.1. Anesthesia and Operative Procedure

**Figure 1:**
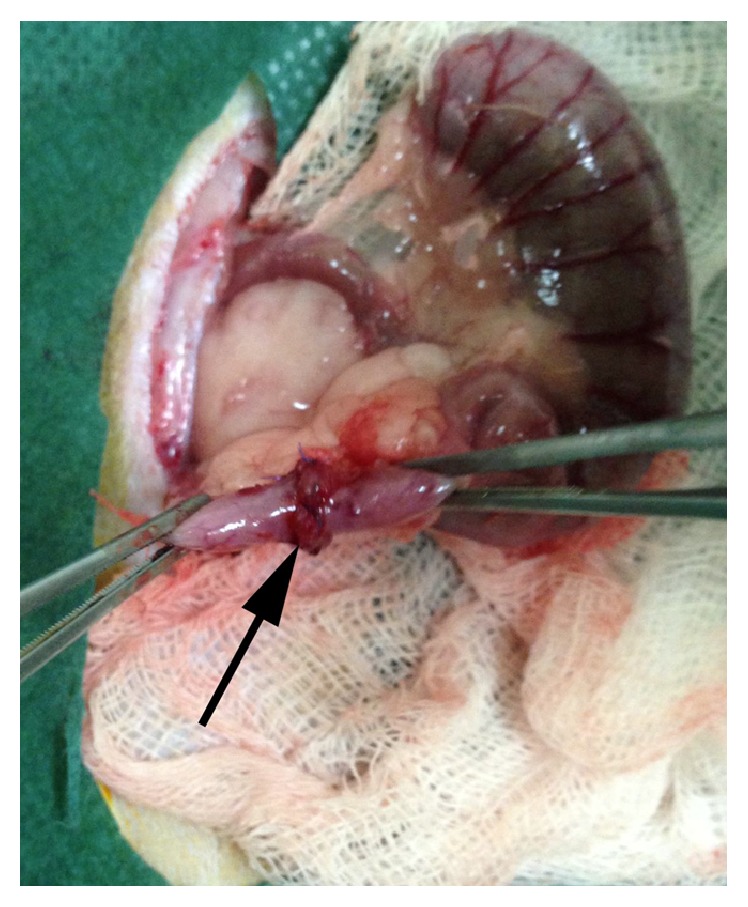
Intraoperative photo, depicting the newly constructed anastomosis (black arrow).

All procedures were carried out under general anesthesia with intramuscular injection of ketamine hydrochloride (50 mg/kg) and xylazine (5 mg/kg). Animals were placed in a supine position, shaved, and disinfected with iodine povidone. A 4 cm midline laparotomy was performed. “Ascending” colon transection, 3 cm distal to the cecum, was carried out and an end-to-end hand sewn anastomosis was constructed by the same operator (GPF) using eight single-layer, everting, interrupted 6/0 monofilament polydioxanone sutures (PDS) (Figure 1). The abdominal wall was closed in a two-layer running fashion using MonoPlus 3/0. The animals were housed individually postoperatively.

### 2.2. Experimental Design

The rats were randomly assigned into two groups with allocation concealment after randomization. Investigators carried out all procedures and measurements blindly until the final data analysis. The experimental group received lornoxicam (4 mg/mL), at a clinically relevant analgesic dose of 1.3 mg/kg per day immediately before and daily after surgery intraperitoneally (i.p.) divided into two doses [14]. The control group received a similar volume of solvent only (0.5 mL N/S). Dosing was continued until the animals were sacrificed. Half the animals in each group were sacrificed on the 3rd POD and the other half on the 7th POD.

### 2.3. Macroscopic Assessment

All animals were euthanized under general anesthesia according to European legislation and ethics for animal care. During postmortem examination, macroscopic assessment of the anastomosis was performed and the peritoneal cavity was explored for signs of anastomotic complications such as generalized peritonitis and abscess formation at the anastomotic site as well as the integrity of the anastomosis and the presence of leakage and/or anastomotic dehiscence. Stenosis was defined as anastomotic diameter less than 50% of the proximal bowel. The formation of adhesions was recorded and evaluated in a blind fashion according to the Knightly scoring scale [15].

### 2.4. Histopathological Examination

The ascending colon was dissected en bloc including the anastomosis along with a 1 cm distal and proximal portion of the bowel and the specimen was placed in 4% formalin solution. Tissue samples were stained with hematoxylin and eosin. An experienced histopathologist carried out histological assessment blindly. Histological variables measured were depth and extent of colon wall destruction. The respective grading is seen in Table 1. Semiquantitative methods were used to evaluate neutrophilic and lymphocytic inflammatory infiltrates, macrophage density, fibroblast in growth, collagen deposition, angiogenesis, and extent and amount of granulation tissue near the anastomotic region by assigning a score ranging between 0 and 3 to each tissue specimen as follows: 0: absent, 1: minimally present, 2: moderately present, and 3: markedly present. Also reepithelization was assessed as follows: 0: none, 1: little, one-layer cubic, 2: much, one-layer cubic, 3: almost complete, one-layer cubic, 4: complete, one-layer cubic, and 5: normal glandular mucosa; similarly necrosis, presence of exudates and microabscesses, and foreign body reaction were noticed.

### 2.5. Statistical Analysis

Differences among groups, with respect to macro- and microscopic indicators of anastomotic healing, were compared using a two-tailed Fisher exact test. When the observed count was less than 5 within a cross tabulation the Monte-Carlo method for calculating the *p* value was used. All reported *p* values were two-tailed with *p* < 0.05 considered as significant [16].

## 3. Results

In total, 4 animals died during the surgical procedure, one in the experimental group and 3 in the control group, due to anesthetic complications. Their data were excluded from the study and the experiment was completed with 12 rats in each group.

### 3.1. Macroscopic Findings

Overall 3 animals from the lornoxicam group developed generalized peritonitis: 2 by 3rd POD and 1 by 7th POD. However, this difference did not attend statistical significance when compared with respective controls (Fisher exact test (Monte-Carlo method), *p* = 0.227 and *p* = 0.500, resp.). Similarly, perianastomotic abscess was seen in 3 animals in the lornoxicam group by the 3rd POD and 1 by 7th POD in comparison with none in the control group, but this difference again was not statistically significant (Fisher exact test (Monte-Carlo method), *p* = 0.091 and *p* = 0.500, resp.). Anastomotic dehiscence was found in two animals in the lornoxicam group both by the 3rd POD, in comparison with none in the control group; however this difference was again not statistically significant (Fisher exact test (Monte-Carlo method), *p* = 0.227). Numerous extensive adhesions were commonly found in both groups but they were not statistically significant (Fisher exact test (Monte-Carlo method), *p* = 0.59). Although anastomotic stenosis was more frequent among the lornoxicam group, no statistical difference was seen between the groups (Fisher exact test (Monte-Carlo method), *p* = 0.59).

### 3.2. Histopathological Examination

Raw data and clustered and overall comparisons between control and experimental group in terms of histologic parameters studied are presented in Table 2; as seen in Table 2 deep reaching defects (depth of colon destruction) attained statistical significance in the lornoxicam group 7th POD. Also subjects treated with lornoxicam had significant lower fibroblast infiltration by the 3rd POD, which was retained in overall comparison. Similarly, subjects treated with lornoxicam had significantly less granulation tissue by 7th POD, but not during the early phase of healing. Surprisingly, inflammatory cell infiltration (polymorphonuclear leukocytes, lymphocytes, and macrophage density) did not differ statistically between groups; however when clustered differences were examined (i.e., by 3rd POD and by 7th POD), macrophage density was increased among subjects treated with lornoxicam. Statistically significant delay in reepithelization was observed in the experimental group (Fisher exact test (Monte-Carlo method), *p* = 0.049) by the 3rd POD which was abolished on the 7th POD; however this difference was not accompanied by a similar difference in collagen deposition, presence of exudates, and foreign body reaction. Although the presence of microabscesses formation was much more pronounced among the lornoxicam treated subjects when compared with control subjects (Fisher exact test (Monte-Carlo method), *p* = 0.001), when subjects were clustered (i.e., 3rd versus 7th POD), this difference was retained only by the 7th POD (Table 2). Similarly, by the 7th POD necrosis was significantly higher in the experimental group (Fisher exact test (Monte-Carlo method), *p* = 0.015), a difference that was not obvious by the 3rd POD.

## 4. Discussion

Anastomotic dehiscence is a much-feared complication after colorectal resections due to consequent increased morbidity and mortality. The usage of NSAIDs has gained ground since their administration has been proven to reduce postoperative pain and opioid consumption [17]. Their pharmacologic action is achieved through inhibition of COX enzymes that catalyze the first step of synthesis of prostanoids [18]. Prostaglandins and thromboxane are involved in inflammatory process and their conversion from arachidonic acid is inhibited by NSAIDs, which has been postulated to adversely affect the inflammatory phase of healing [19, 20]. Furthermore, a well-recognized complication of NSAIDs usage is ulceration and bleeding from the gastrointestinal mucosa, including the colonic mucosa [21, 22]. These adverse effects may in theory represent a potential risk for the inflamed mucosa in a new anastomosis.

A current review of the literature revealed more than three observational clinical studies reporting on a higher occurrence of postoperative anastomotic leakage with the use of NSAIDs [13, 23]. Although the majority of available studies have an inclined trend towards more leaks among NSAIDs treated patients compared to controls, two of these prospective clinical studies failed to confirm this trend [13]. Though there is no evidence to support an increased risk of anastomotic leak with NSAID's administration, the most recent meta-analysis has postulated that the true risk from NSAID administration cannot be reliably evaluated within a clinical setting due to multiple cofounding factors and their possible cumulative effect, including level of rectal anastomosis, bowel preparation, defunctioning loop ileostomy, epidural anesthesia, and comorbidities [3, 4, 13, 24, 25]. Klein et al. have suggested that “an optimal and adequately powered study design would have been a randomised controlled trial including 2100 patients in each group to identify a decrease in risk of 30 % in anastomotic leakage. Setting up such a large RCT may prove impossible, or at best, challenging” [26].

The proposed pathophysiologic mechanism by which NSAIDs may adversely affect anastomotic healing is related to COX-2 inhibition [27–29]. COX-2 is strongly upregulated during the early postoperative period after intestinal anastomosis [27]. It is well known that COX-2 inhibition adversely affects the healing of soft tissue injuries and fractures [28–30]. It has been shown that COX-2 selective NSAIDs, celecoxib and diclofenac, increased the anastomotic leakage rate while nonselective ibuprofen did not [23]. On the contrary, a study by Schlacta et al. revealed high, though nonsignificant, leakage rate with a COX-1 selective NSAID [31]. NSAIDs exhibit significant differences in regard to pharmacokinetic parameters such as rate of absorption, bioavailability, and elimination half-life, as well as differences in metabolism and drug interactions, and thus may exert their adverse effects on anastomotic healing, by more than a single mode of action [27]. Lornoxicam is a potent nonselective inhibitor of the COX enzymes but unlike some NSAIDs it inhibits both COX enzymes in the same concentration range; that is, COX-1/COX-2 = 1 [32].

Mucosal integrity has long been recognized to be dependent on COX-1 derived prostaglandins [33]. Deep ulceration occurring at the anastomotic site may adversely affect the integrity of a newly constructed anastomosis. COX inhibitors may also affect the inflammatory cell infiltration of the anastomotic site. It has been supported that COX-2 promote the resolution of the inflammatory response, by decreasing the necessary chemotactic factors for infiltration by leucocytes [30], albeit, in our study, we failed to notice a significant difference in the inflammatory infiltration around the anastomosis among lornoxicam treated subjects. Furthermore, COX inhibitors are known to affect the neoangiogenesis, an important element of healing process [34, 35]. Fibroblast activity and granulation tissue may be the most important factors affecting the anastomotic integrity due to their contribution to collagen production, which is the most important molecule for anastomotic strength [27, 34, 36]. Exogenous administration of prostaglandins has been shown to increase the amount of early anastomotic collagen content and thus anastomotic strength [37, 38]. Our results have shown decreased fibroblast infiltration and granulation tissue among lornoxicam treated subjects, which in turn may explain the increased occurrence of anastomotic leak observed. In addition, NSAIDs may affect anastomotic healing by decreasing the blood supply to the anastomosis secondary to microthrombosis or microemboli, known to occur with NSAID's administration [7]. This ischemic effect of NSAIDs may be the explanatory mechanism for the increased necrosis seen in proximity to the anastomosis in our study. NSAIDs could inhibit superoxide anion generation, which in turn could result in decreasing production of oxygen-derived radicals and hence increased formation of micro- or macroabscesses, as seen in the present study [39].

Although NSAIDs affect various histologic variables, the most important net result, that is, anastomotic dehiscence, may be associated with other confounding factors that cannot be accounted for. Despite the limitations of the present study, namely, the small sample size, perioperative administration of lornoxicam appears to impair colonic anastomotic healing and to our knowledge, this is the only study evaluating the effects of intraperitoneal lornoxicam administration.

## 5. Conclusions

In our study, although perioperative administration of lornoxicam did not induce significant increase of anastomotic dehiscence in a rat model, histological parameters associated with the healing process of a newly reconstructed colonic anastomosis were shown to be impaired. In the light of the results of our study, deep reaching defects and delayed reepithelization in the experimental group indicate a trend towards more leaks. However, use of NSAIDs remains an extremely useful analgesic adjunct. Therefore, further studies are needed before compelling evidence accumulates on the effects of available COX-1 and COX-2 inhibitors on intestinal anastomotic healing and their role following colorectal anastomosis. Until such evidence is available, caution should be exercised in the perioperative use of lornoxicam and other NSAIDs in gastrointestinal surgery.

## Figures and Tables

**Table 1:** Grading score of depth and extent of colonic wall destruction.

Depth of colonic wall destruction (affected layers)	Grade	Extent of colonic wall destruction	Grade
None	0	No damage	0
Mucosal erosion	1	Focal lesions (<20%)	1
Submucosal erosion	2	Localized lesions (<40%)	2
Ulceration reaching muscularis propria	3	Widespread lesions (>40%)	3
Ulceration reaching adventitia	4		

**Table 2:** Raw data and clustered and overall comparisons of histologic variables studied between controls and lornoxicam treated subjects. Comparisons were made with Fischer exact test. Variables with statistically significant differences are presented.

	Grading score	CG3 *n* = 6	EG3 *n* = 6	*p* value	CG7 *n* = 6	EG7 *n* = 6	*p* value	Overall
								*p* value
								CG versus EG
Depth of colon destruction	2	1/6	0/12	0.270	0/6	0/6	0.015^*∗*^	0.009^*∗*^
	3	5/6	5/6		6/6	1/6		
	4	0/6	1/6		0/6	5/6		

Fibroblast infiltration	1	0/6	4/6	0.023^*∗*^	0/6	2/6	0.540	0.015^*∗*^
	2	5/6	2/6		4/6	3/6		
	3	1/6	0/6		2/6	1/6		

Granulation tissue	1	4/6	5/6	1	0/6	4/6	0.002^*∗*^	0.005^*∗*^
	2	1/6	0/6		6/6	0/6		
	3	1/6	1/6		0/6	2/6		

Macrophage density	1	5/6	5/6	1	5/6	0/6	0.015^*∗*^	0.082
	2	1/6	1/6		0/6	3/6		
	3	0/6	0/6		1/6	3/6		

Reepithelization	0	0/6	4/6	0.005^*∗*^	0/6	0/6	1	0.049^*∗*^
	1	0/6	2/6		0/6	0/6		
	2	2/6	0/6		2/6	3/6		
	3	4/6	0/6		3/6	3/6		
	4	0/6	0/6		1/6	0/6		

Microabscesses	Presence	1/6	5/6	0.08	0/6	5/6	0.015^*∗*^	0.01^*∗*^

Necrosis	0	2/6	2/6	0.474	1/6	0/6	0.015^*∗*^	0.003^*∗*^
	1	4/6	2/6		5/6	1/6		
	2	0/6	2/6		0/6	5/6		

CG3: control group 3rd POD, EG3: experimental (lornoxicam) group 3rd POD, CG7: control group 7th POD, EG7: experimental (lornoxicam) group 7th POD, and *n*: number of subjects.

^*∗*^Statistical significance at the level of 0.05.
